# In Situ Formation of Nanoparticles from Graft Copolypeptides Under Dispersion Polymerization Conditions

**DOI:** 10.1002/marc.202500069

**Published:** 2025-04-28

**Authors:** Ernesto Tinajero‐Díaz, Robert D. Murphy, Bo Li, Andreas Heise, Antxon Martínez de Ilarduya

**Affiliations:** ^1^ Departament d'Enginyeria Química Universitat Politècnica de Catalunya, ETSEIB Diagonal 647 Barcelona 08028 Spain; ^2^ Department of Chemistry RCSI University of Medicine and Health Sciences 123 St Stephens Green, Dublin 2 Dublin Ireland; ^3^ Science Foundation Ireland (SFI) Centre for Research in Medical Devices (CURAM) RCSI Dublin 2 Dublin Ireland; ^4^ AMBER The SFI Advanced Materials and Bioengineering Research Centre RCSI Dublin 2 Dublin Ireland

**Keywords:** dispersion polymerization, polypeptides, ring‐opening polymerization, α‐amino acid N‐carboxyanhydrides, ε‐poly(lysine)

## Abstract

A simple method is presented for preparing polypeptide nanoparticles using hydrophilic biosynthetic ε‐poly(lysine) (εPL) as a reactive surfactant under dispersion polymerization conditions. In situ graft polymerization of benzyl‐L‐glutamic acid *N*‐carboxyanhydride (BLG‐NCA) triggers the self‐assembly of amphiphilic copolymers into nanoparticles, which are colloidally stabilized by the remaining εPL amino groups at the particle surface. The average nanoparticle diameter can be controlled in the range of 40–120 nm by varying the initiator‐to‐NCA ratio, as demonstrated by the correlation between graft copolymer molecular weight (measured by size exclusion chromatography) and the z‐average diameter (measured by dynamic light scattering). Secondary structure analysis indicates that the α‐helical conformation of poly(benzyl‐L‐glutamate) (PBLG) grafts plays a role in both accelerating NCA polymerization and stabilizing the nanostructures. This approach is readily scalable to high concentrations and offers a straightforward route to peptidomimetic nanoparticles, entirely composed of amino acids, with promising potential for nanomedicine applications.

## Graphical abstract

Biosynthetic ε‐polylysine acts as macroinitiator in the ring‐opening dispersion polymerization of α‐amino acid *N*‐carboxyanhydrides (NCAs) triggering graft copolymer self‐assembly into polypeptide nanoparticles. The nanoparticle sizes can be tuned by modulating the side‐chain length while retaining the helical secondary structures of the polypeptide in the nanoparticle core.

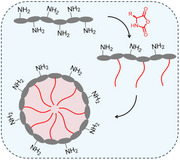

## 1 Introduction

Nanoparticles derived from natural or nature‐inspired polymers play a pivotal role in advancing nanomedicine, owing to their biodegradability and inherent biocompatibility.^[^ ^1^, ^2^, ^3^, ^4^ ^]^ Synthetic polypeptides, as simplified peptide mimics, hold considerable promise in this regard. These polymers are readily synthesized via controlled ring‐opening polymerization (ROP) of α‐amino acid *N*‐carboxyanhydrides (NCAs) using primary amine initiators.^[^ ^5^, ^6^, ^7^, ^8^ ^]^ Several strategies have been explored for fabricating polypeptide nanoparticles, with the self‐assembly of amphiphilic block copolypeptides into micelles or vesicles being the most common approach.^[^ ^9^, ^10^, ^11^, ^12^, ^13^ ^]^ However, this method can be labor‐intensive, requiring precise control over solvent exchange conditions. More recently, the formation of polypeptide nanoparticles through mini‐emulsion polymerization has been reported.^[^ ^14^, ^15^ ^]^ This technique involves the polymerization of water‐sensitive NCAs within surfactant‐stabilized oil droplets in the aqueous phase. While straightforward, this method has so far only been demonstrated with complex polypeptide surfactants. Another widely used approach in radical polymerization for nanoparticle synthesis is dispersion polymerization in aqueous media,^[^ ^16^, ^17^, ^18^, ^19^ ^]^ though it remains largely unexplored for polypeptide nanoparticles. In this process, a soluble monomer is polymerized in the presence of an amphiphilic surfactant. As the polymerization progresses, the polymer becomes insoluble and forms particles. In some cases, the surfactant also acts as the initiator, generating the amphiphilic species in situ during the early stages of the reaction. A special variation of dispersion polymerization, known as Polymerization‐Induced Self‐Assembly (PISA), has drawn attention due to its ability to control nanoparticle morphology.^[^ ^20^, ^21^, ^22^, ^23^, ^24^ ^]^ In PISA, chain extension from a soluble macromolecular precursor results in the formation of amphiphilic block copolymers, which self‐assemble into nanoparticle colloids once a critical degree of polymerization (DP) is reached.^[^ ^25^ ^]^ Throughout this process, the chain ends remain active, allowing for further chain extension and enabling the morphological phase transition of the nanoparticles as the block length ratio evolves. Several recent studies have explored NCA‐based PISA techniques, with amino‐terminated hydrophilic macroinitiators like poly(ethylene glycol) (PEG) and poly(sarcosine) being utilized in aqueous NCA ROPISA.^[^ ^26^, ^27^, ^28^, ^29^, ^30^, ^31^, ^32^, ^33^ ^]^ We have also recently reported a procedure in which both blocks are polymerized sequentially in a single solvent system (acetonitrile/water, 1:1), leading to the formation of spherical and elongated nanoparticles.^[^ ^34^ ^]^ However, one limitation of NCA PISA is the complexity of process optimization and the limited availability of suitable macroinitiators. If only spherical nanoparticles are desired, a more straightforward and robust method, akin to conventional dispersion polymerization, would be preferable. A surfactant frequently used in the food industry is ε‐polylysine (εPL), a cationic, naturally occurring homopolyamide of L‐lysine, produced by *Streptomyces albulus*.^[^ ^35^, ^36^, ^37^ ^]^ εPL is water‐soluble, biodegradable, edible, and non‐toxic, making it ideally suitable as an easily accessible renewable and biocompatible emulsifying agent. In this study, we demonstrate the use of biosynthetic εPL as a hydrophilic polypeptide surfactant and initiator for the formation of spherical nanoparticles under dispersion polymerization conditions. This represents the first reported use of a side‐chain polyamine in NCA ring‐opening polymerization‐induced self‐assembly and is conceptually distinct from the chain‐end extension strategies previously reported.

## 2 Results and Discussion

ε‐Polylysine (εPL) was produced through microbial fermentation, yielding a polymer with an average of 24 lysine residues (*M*_n_ = 3090 g mol^−1^) (Figure S1, Supporting Information). Based on our previous work, acetonitrile/water (ACN/water) was selected as the reaction medium for the polymerization of benzyl‐L‐glutamate *N*‐carboxyanhydride (BLG‐NCA).^[^ ^34^ ^]^ The selection of the ACN/water mixture was based on the following criteria: i) water acts as the most prevalent proton transfer catalyst and facilitates the process through H‐bond networks composed of multiple water molecules, thus, accelerating the ROP of α‐amino acids NCA,^[^ ^38^, ^39^ ^]^ ii) the change in solvent polarity and/or dielectric constant (water = 80.1; acetonitrile = 37.5) in the binary ACN/water mixture may also contribute to accelerating the ROP of NCA, while simultaneously enhancing the solubilization of both the macroinitiator and the NCA monomer. However, this effect is expected to be more pronounced for the latter, given the inherently poor water solubility of the NCA monomers employed, and iii) given the εPL insolubility in acetonitrile, a water content of 40–60% was optimal, as lower water concentrations would be insufficient to fully dissolve it. Additionally, an excess of water would negatively impact polymerization control by increasing competition from monomer hydrolysis.

Accordingly, to ascertain optimal solvent conditions, controlled experiments were conducted using different percentage of water content for the ROP of BLG‐NCA initiated by εPL ([M]:[I] = 6) and a solid content (σ_s_) of 1.5% (Figure S2, Supporting Information). SEC analysis of the copolypeptides showed monomodal peaks and reproducible *M*_n_’s along the whole range of solvent compositions. However, the 1:1 v/v mixture provided the narrowest dispersity (*Đ* = 1.15), and we selected this solvent composition for further NCA polymerizations.

**Figure 1:**
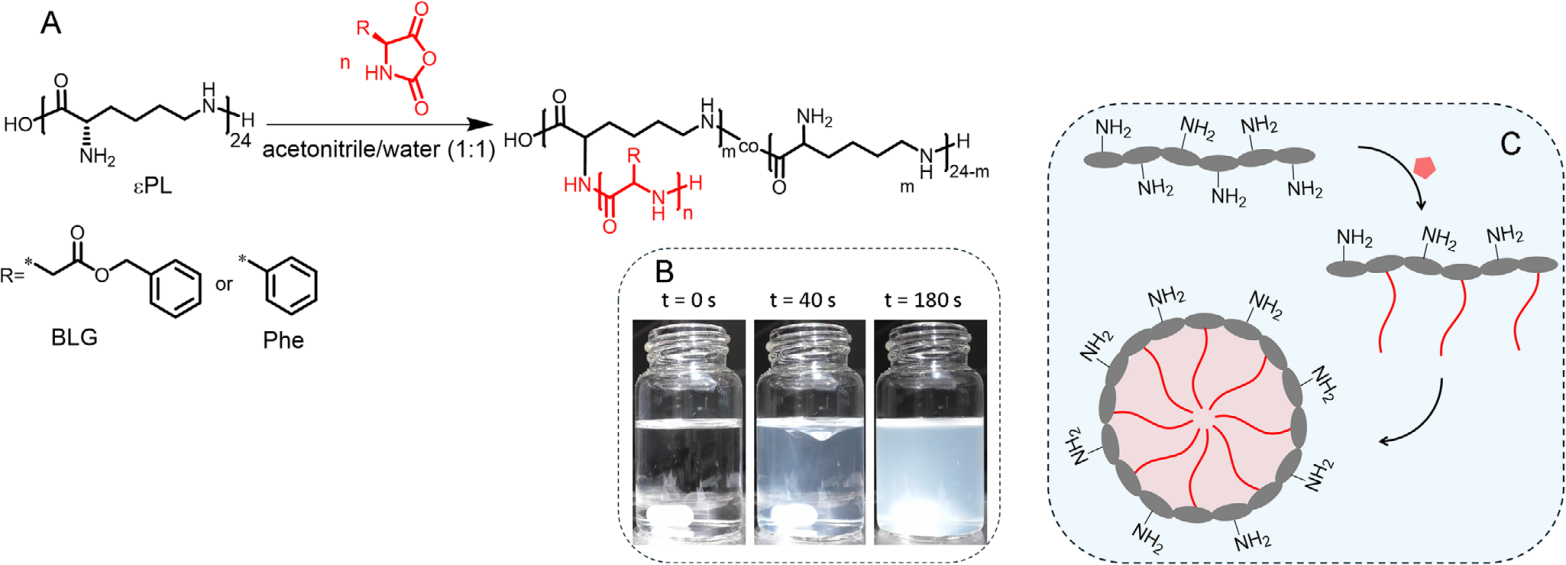
Preparation of nanoparticles by NCA graft copolymerization from εPL under dispersion polymerization conditions. A) synthetic reaction scheme; B) images of BLG‐NCA solutions in ACN/water (1:1) at different time points after εPL addition; C) proposed pathway of in situ nanoparticle formation in ACN/water (1:1) solvent mixture.

Thereof, we hypothesized that adding εPL to a solution of BLG‐NCA in ACN/water would initiate the NCA ring‐opening polymerization through some of the amine groups of εPL, resulting in the in situ formation of amphiphilic graft copolypeptides (**Figure** **1**A). These graft copolymers would then self‐assemble, stabilizing a colloidal dispersion of poly(benzyl‐L‐glutamate) (PBLG) nanoparticles (Figure 1B). Before attempting the polymerization, initial experiments confirmed the hydrolytic stability of BLG‐NCA in this solvent mixture, with no evidence of monomer hydrolysis or polymerization detected by ^1^H NMR spectroscopy for up to 11 min (Figure S3, Supporting Information).

**Figure 2:**
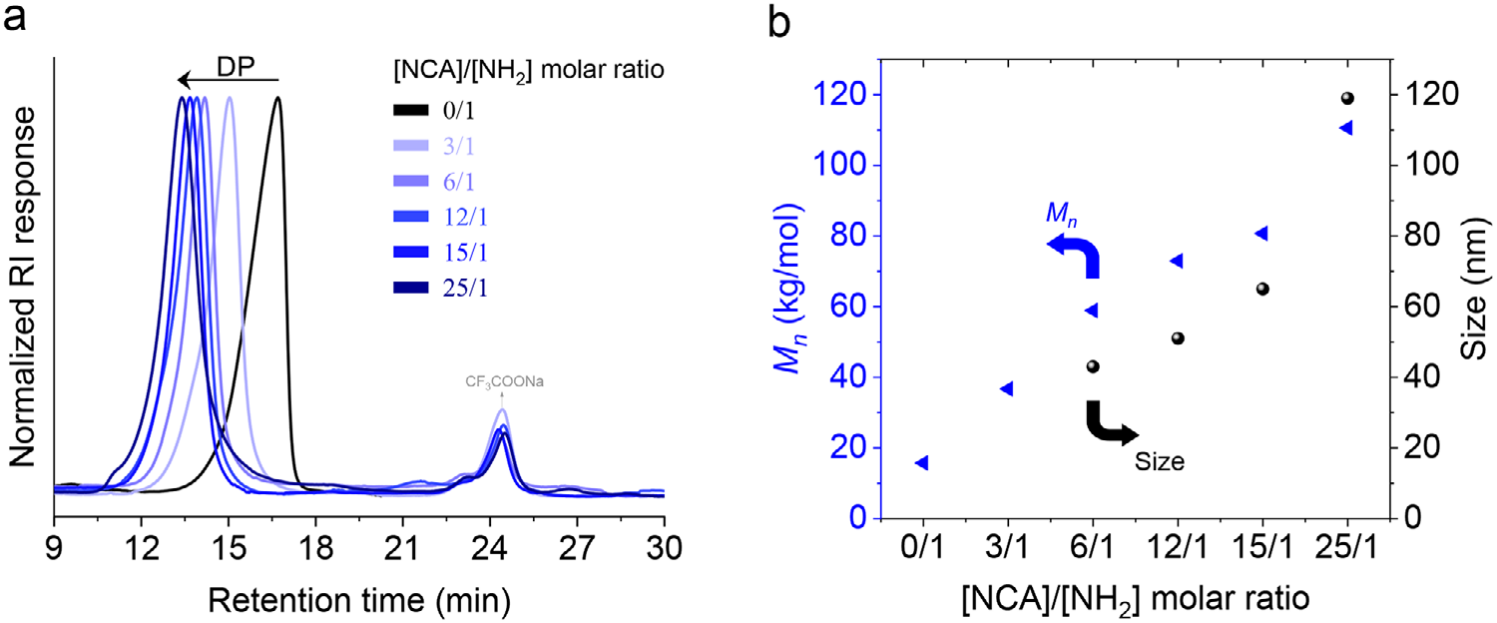
a) Evolution of SEC traces with increasing BLG‐NCA to εPL‐amine molar ratio; b) SEC number‐average molecular weights (*M*_n_) and DLS z‐average diameters as a function of the εPL‐amine to BLG‐NCA molar ratio.

A series of experiments were then conducted with varying ratios of εPL‐amines to BLG‐NCA (1:3, 1:6, 1:9, 1:12, and 1:24) at a solid content (*σ*_s_) of 1.5% (**Table** **1**, entries 2–6). When a solution of εPL was added to the NCA solutions, a transition from a clear to a turbid bluish solution was observed within 10 min for all samples, indicating the formation of nanoparticles (Figure 1B; Video S1, Supporting Information), except for the 1:3 ratio sample, which precipitated. Thus, we withdrew an aliquot directly from the reaction medium for FTIR analysis, which confirmed the complete consumption of BLG‐NCA. This was evidenced by the disappearance of the characteristic carbonyl infrared bands of the NCA anhydride groups at 1789 and 1850 cm⁻¹. For the 1:6 εPL‐amines to BLG‐NCA ratio, ¹H NMR spectra at various time intervals showed a decrease in the signals for the benzyl (−CH₂Ar−) and aryl (−CH₅−) protons at 5.4 and 7.7 ppm, respectively (Figure S4a, Supporting Information). Additionally, we explored the grafting reaction at different reaction times and analyzed the copolypeptide after precipitation in methanol. Interestingly, the composition matched the initial feed ratio (Figure S4b, Supporting Information). This observation further corroborates the FTIR results and confirms complete monomer conversion, thus marking the end of the reaction at 11 min. Final samples from all reactions were lyophilized and analyzed by size‐exclusion chromatography (SEC), which revealed monomodal traces and low dispersities for all polymers. Notably, a clear shift in the SEC traces to higher molecular weights was observed with increasing NCA to εPL ratios, with no residual εPL detected in the chromatograms (**Figure** **2**a). These results suggest that εPL acts as a macroinitiator, facilitating the formation of graft copolymers with PBLG chains grafted from the amino groups of εPL. Given the heterogeneity of the reaction, the nearly linear increase in the number‐average molecular weight (*M*_n_) with the NCA to εPL ratio was surprising but may indicate a high degree of control in this graft copolymerization process (Figure 2b). To further confirm the chemical constitution of the graft copolypeptide, we performed a DOSY NMR analysis over freeze dried samples. The DOSY spectra of εPL_24_‐*g*‐PBLG_6_ is shown as representative polymer in Figure S5 (Supporting Information).

**Table 1:** Results of the in situ formation of nanoparticles from graft εPL copolypeptides.

Entry	Sample	[NCA]/[NH_2_]^a)^	*M*_n_ ^b)^ (g·mol^−1^)	*Đ* ^b)^	d_hydr_ ^c)^ [nm]	PDI^c)^	ζ^c)^ [mV]
1	εPL_24_	0/1	15 700	1.17	−	−	−
2	εPL_24_‐*g*‐PBLG_3_	3/1	36 700	1.23	−	−	−
3	εPL_24_‐*g*‐PBLG_6_	6/1	58 900	1.15	43	0.26	38
4	εPL_24_‐*g*‐PBLG_12_	12/1	73 000	1.24	51	0.23	54
5	εPL_24_‐*g*‐PBLG_15_	15/1	80 700	1.17	65	0.17	37
6	εPL_24_‐g‐PBLG_25_	25/1	110 600	1.43	119	0.23	16
7	εPL_24_‐*g*‐Phe_5_	5/1	40 800	1.12	47	0.14	35

^a)^ Experimental molar ratio, i.e., DP of the poly(α‐amino acid) segment determined by ^1^H NMR;

^b)^ SEC;

^c)^ DLS.

Signals from the different monomeric units exhibit a similar diffusion coefficient (*D*) of 1.13 × 10^−10^ m^2^ s^−1^ corroborating the existence of a single macromolecule. For reference, the DOSY spectrum of εPL is also shown, with a *D* = 0.32 × 10^−10^ m^2^ s^−1^. The lower D value may result from repulsion between adjacent ionic charges as the spectrum was recorded under acidic conditions, where the amine side groups are then protonated, leading to a more extended chain conformation.^[^ ^40^ ^]^ Thus, results from SEC, along with spectroscopy analyses, demonstrate the successful synthesis of the graft copolypeptide.

**Figure 3:**
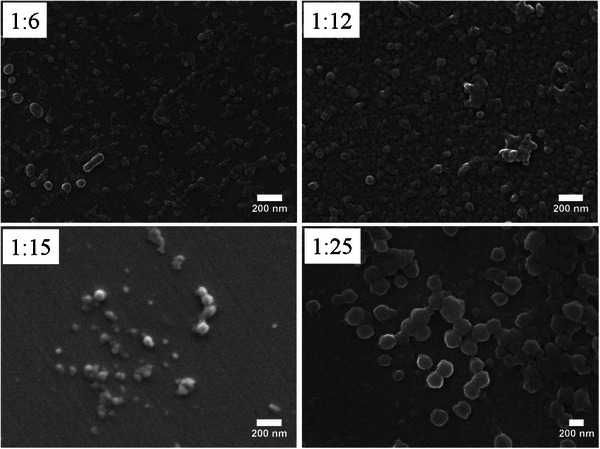
SEM images of the morphologies seen under dispersion polymerization of BLG‐NCA. Scale bar = 200 nm.

For εPL to BLG‐NCA ratios ranging from 1:6 to 1:24, dynamic light scattering (DLS) measurements revealed z‐average diameters between 43 and 119 nm, with polydispersity indices (PDI) ranging from 0.17 to 0.26. The increase in nanoparticle size appears to correlate directly with the molecular weight of the graft copolypeptides (Figure 2b), although additional data points are needed to draw a definitive conclusion. Over a four‐week period, the z‐average size showed only a moderate increase in both size and polydispersity, indicating that the system exhibits high colloidal stability. However, when acetonitrile was removed by dialysis, the nanoparticles precipitated, suggesting that εPL chains alone were unable to stabilize the non‐solvated PBLG chains in water. Scanning electron microscopy (SEM) and transmission electron microscopy (TEM) images were recorded for all samples (**Figure** **3**; Figure S5, Supporting Information). Both techniques revealed that the nanoparticles generally exhibited a spherical morphology, with particle diameters in good agreement with those determined by DLS. However, some samples displayed a population of particles with more irregular shapes, as well as particle clustering, particularly evident in the TEM images of samples prepared from εPL‐amine to BLG‐NCA ratios of 1:6 and 1:12. This clustering is believed to result from sample preparation processes, including the evaporation of lower‐boiling ACN, and is consistent with the particle aggregation observed when ACN was removed via dialysis.

Given the pronounced hydrophobicity of PBLG, it is reasonable to propose that the copolypeptides self‐assemble into a core–shell structure, with PBLG chains forming the core of the particles (Figure 1B). In this model, some amino groups of εPL would initiate the graft polymerization, resulting in the in situ formation of an amphiphilic graft copolypeptide that drives self‐assembly. The observed positive zeta potential values (ζ), ranging from +16 to +54 mV, can be attributed to the presence of free protonated amino groups from εPL exposed on the nanoparticle surface. These surface‐exposed primary amines are positively charged under the experimental conditions, resulting in a net positive surface potential. The relatively high ζ‐potential values further strengthen the good colloidal stability, likely due to electrostatic repulsion between nanoparticles in the dispersion medium (Table 1; Figure S7, Supporting Information).^[^ ^41^ ^]^

**Figure 4:**
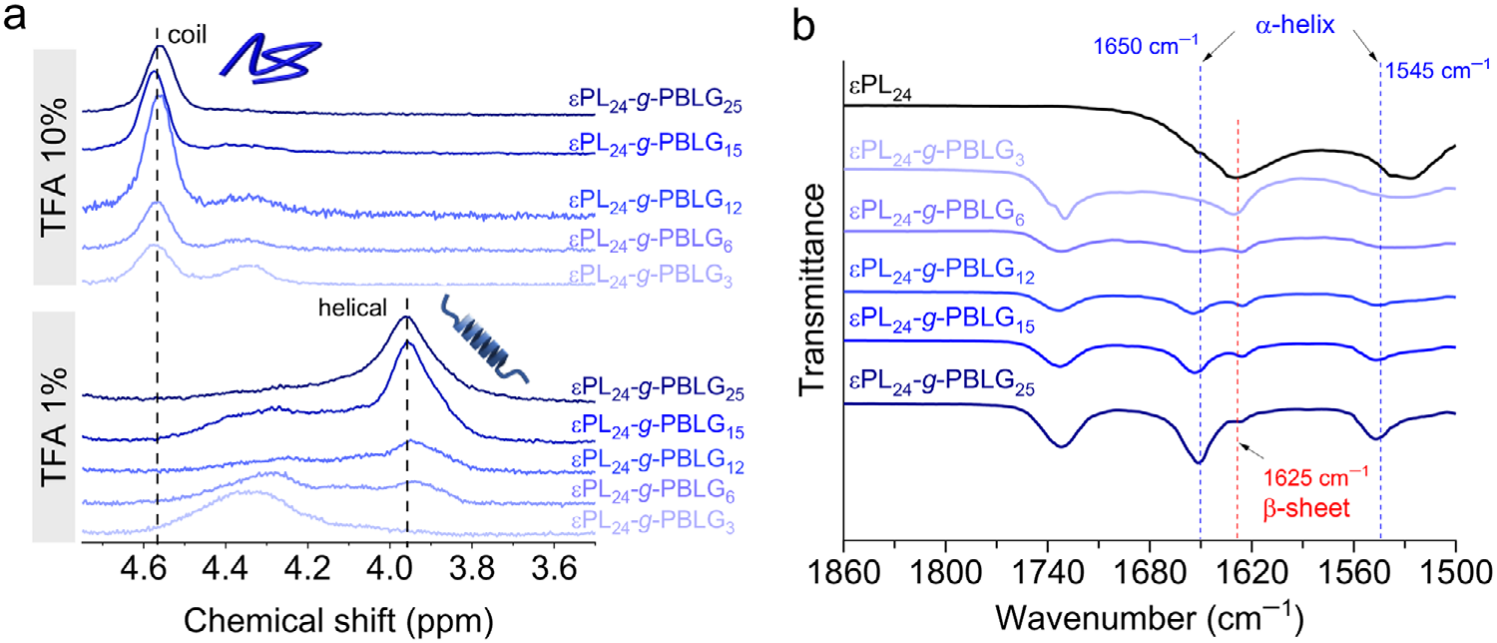
Spectra region of the β‐sheet and α‐helical secondary structures of the εPL_24_‐*g*‐PBLG_m_ copolypeptides in solution and solid state: a) ^1^H NMR in CDCl_3_–TFA (1 and 10% v/v). For ease of interpretation, we omitted spectra at intermediate TFA concentrations, and b) ATR‐FTIR.

We hypothesize that the grafting process is likely governed by a cooperative polymerization effect, where the proximity of α‐helices formed by PBLG side chains may enhance the polymerization kinetics in a self‐catalyzing manner, as demonstrated by Chen et al. in their work on dendritic initiation.^[^ ^42^, ^43^ ^]^ This process would favor chain propagation from PBLG grafts in the confined space of a self‐assembled particle over additional initiation from free εPL groups amino at an early stage of the reaction thus leaving some εPL amino groups unreacted. Supporting evidence for the contribution to the confined polymerization environment was obtained from a control experiment in which BLG‐NCA was initiated by εPL in a homogenous medium, i.e., in ACN/water (1:1 v/v). For a molar ratio of εPL to BLG‐NCA of at least 1:3 the ^1^H NMR signal of the CH proton at 4.14 ppm adjacent to the amino group disappeared, suggesting quantitative initiation from the εPL amino groups (Figure S8, Supporting Information). Further evidence for the contribution of secondary structure to the copolypeptide assemblies was obtained through ¹H NMR and FTIR analysis of the nanoparticles in both solution and solid‐state forms. For the ¹H NMR analysis, portions of trifluoroacetic acid (TFA) were successively added to freeze‐dried copolypeptides in CDCl_3_, with concentrations ranging from 1% to 10% (v/v). While the copolypeptide was insoluble in pure CDCl_3_, the addition of small amounts of TFA resulted in well‐resolved spectra. Upon adding 1% TFA, a signal at 3.95 ppm, corresponding to the α‐CH protons of PBLG, appeared, indicating the presence of an ordered helical structure.^[^ ^44^, ^45^, ^46^ ^]^ As the TFA concentration increased, this signal gradually shifted, reflecting an α‐helix to random coil transition. At an εPL‐amine to BLG‐NCA ratio of 1:3, the side chains were too short to stabilize an α‐helix, and no characteristic resonance was observed. However, as the ratio increased, the α‐helix resonance at 3.95 ppm became more prominent, confirming the presence of α‐helical structures (**Figure** **4**a). With higher TFA concentrations (2–10%), the signals corresponding to the ordered helical state gradually diminished, eventually disappearing, while a new resonance at 4.58 ppm emerged, indicating the formation of a non‐ordered (random coil) conformation and signifying a complete disruption of the helical structure. To further investigate the thermal stability of the helix, we heated the copolypeptide solutions, inducing the inverse transition from random coil to helix within the 25–60 °C range. This treatment resulted in nearly complete conversion to the helical conformation for the εPL‐amine to BLG‐NCA ratio of 1:25, while copolypeptides with ratios of 1:15 and 1:12 showed moderate restoration of the helical structure. No conformational transition was observed for the 1:6 sample, even at 60 °C for the copolypeptides with ratios of 1:6 (Figure S9, Supporting Information). This inverse thermal transition is explained by the entropy gain of TFA molecules interacting with the polypeptide backbone, which disrupts the original structure, alongside enthalpic changes due to alterations in hydrogen bonding interactions.^[^ ^47^ ^]^

A similar trend to that observed in solution was also evident in the bulk state at 25 °C. Infrared spectra of freeze‐dried samples were recorded and analyzed, with a focus on the Amide I and II regions (Figure 4b). The spectrum of the copolypeptide εPL‐amine to BLG‐NCA ratio of 1:3 primarily exhibited characteristic bands corresponding to the β‐sheet conformation. However, as the PBLG block length increased, distinct infrared bands associated with the α‐helical conformation emerged. Through deconvolution of the spectra, the α‐helix content was quantified, ranging from 66% to 89% (Figure S10, Supporting Information). These results suggest that as the PBLG block length increases, the α‐helix becomes more prominent as expected. Notably, the trends observed in the bulk state align closely with those found in solution, collectively supporting the conclusion that the side chains predominantly adopt an α‐helical conformation, which can potentially facilitate the proposed accelerated polymerization.^[^ ^42^ ^]^ It might also contribute to the self‐assembly process and particle core stabilization though the alignment of helical segments as previously proposed for PBLG particles.^[^ ^48^, ^49^, ^50^ ^]^

Finally, to provide preliminary evidence for the robustness of the methodology, we increased the solid content *σ*_s_ up to 7% for the εPL‐amine to BLG‐NCA ratio of 1:6. z‐Average diameters ranging from 30 to 76 nm and polydispersity lower than 0.12 (DLS) and monomodal SEC traces with *M*_n_’s ranging between 43 600 and 57 100 g mol^−1^ (Figure S11, Supporting Information). In another experiment, the versatility of the dispersion polymerization reaction using Phenylalanine (Phe) NCA was achieved with similar results as for BLG‐NCA (Table 1, entry 7). Amino groups in εPL successfully triggered the NCA polymerization under similar dispersion conditions (Figures S12 and S13, Supporting Information).

## 3 Conclusion

We present a simple protocol, scalable to high concentrations, for the preparation of polypeptide nanoparticles (NPs) in an open‐to‐air, one‐pot reaction under dispersion polymerization conditions using biosynthetic εPL. In this method, initiation of polypeptide grafting from the amino groups of εPL leads to the formation of stable nanoparticles through self‐assembly of the polypeptides. The unreacted amino groups on εPL at the particle–solvent interface help stabilize the nanoparticles. The size of the NPs can be tuned by adjusting the length of the polypeptide segment. This approach offers several advantages over methods such as solvent exchange, particularly in terms of its simplicity and scalability. It is especially useful for applications where synthetic polymers are not desired and some variation in particle size is acceptable. Future efforts will focus on improving the water dispersibility of the nanoparticles.

## 4 Experimental Section

### Materials

γ‐Benzyl‐L glutamate (BLG) (>99.0%), L‐phenylalanine (Phe) (95.0%), epichlorohydrin (ECH) (>99.0%) and triphosgene (99.0%) were acquired from Fluorochem Ltd. Epsilon‐poly(lysine) (εPL) was kindly gifted from DKSH Marketing Services (Spain). Acetonitrile (ACN) (>99.9%), tetrahydrofuran (THF) (>99.9), ethyl acetate (>99.5%), trifluoroacetic acid (TFA) (>99.0%) were acquired from Sigma–Aldrich. Chloroform‐d (CDCl_3_) and deuterium oxide (D_2_O) were purchased from Euroisotop. Unless otherwise noted, all reagents and chemicals were used as received without further purification.

### Methods

Nuclear magnetic resonance (NMR) spectra were recorded on a Bruker AMX 300.1 MHz (^1^H) as stated in the recorded spectra. All chemical shifts are reported in parts per million (ppm) relative to the reference signal of the tetramethylsilane (TMS). DOSY ^1^H NMR were recorded on a Bruker Avance 400 (400 MHz) spectrometer at room temperature using a CDCl_3_/TFA‐d mixture as solvent. Attenuated total reflection (ATR) FTIR measurements were performed on a Perkin Elmer Frontier FTIR spectrometer. Spectra were obtained from 8 scans with a resolution of 2 cm^−1^ in the spectral region of 4000−650 cm^−1^. Molecular weight analyses of polymers were performed by SEC on a Waters instrument provided with RI and UV detectors. A 100 µL portion of 0.1% (w/v) sample solution was injected and chromatographed using 1,1,1,3,3.3‐hexafluoro‐2‐propanol (HFIP) mixed with sodium trifluoroacetate (5 mm) as a mobile phase with a flow of 0.5 mL·min^−1^. HR5E and HR2 Waters linear Styragel columns (7.8 mm × 300 mm, pore size 103−104 Å) packed with cross‐linked polystyrene and protected with a precolumn were used. Average molar masses and dispersities were calculated against poly(methyl methacrylate) (PMMA) standards. Dynamic light scattering (DLS) analyses were carried out using a Malvern Zetasizer Nano ZSP instrument (Malvern Instruments, Malvern UK) with a detection angle of 173° and a 3 mW He−Ne laser operating at a wavelength of 633 nm. 10 µL dispersed in 1.5 mL of deionized water of solution was used to determine size and distribution of the particles obtained. Transmission Electron Microscopy (TEM) images were recorded on a Hitachi 7650 microscope working at 120 kV. Samples were prepared by spraying a 1 mg·L^−1^ solution of the copolypeptide onto a copper grid (200 mesh, carbon coated), dripping the water excess, and applying negative staining with an 1% phosphotungstic acid (PTA) in water. Scanning Electron Microscopy (SEM) were taken with a field‐emission JEOL JSM‐7001F (JEOL, Tokyo, Japan) from platinum/palladium coated samples. Different dilutions were essayed to observe free individual nanoparticles. Images were edited using the ImageJ software.

### Synthesis of the (Epsilon‐Poly(lysine)_24_‐g‐poly(α‐Amino Acids))

As an example, for the synthesis of (εPL_24_‐*g*‐PBLG_6_) copolypeptide, BLG‐NCA (49.0 mg, 0.19 mmol) was dissolved in 3.3 mL of (ACN:water) (1:1 v/v) under magnetic stirring in a glass vial. Subsequently, 160 µL of a solution of epsilon‐poly(lysine) (εPL) (4 mg, 1.29 mmol) in ACN:water was added into the flask ([M]_0_/[I]_0_ = 6, per every −NH_2_ side chain in εPL). The reaction was left to stir until the BLG‐NCA had been completely consumed as monitored by ATR FTIR spectroscopy. After full monomer conversion, the reaction mixture was dialyzed against water (MWCO = 6.0 kDa) for 24 h.

## Conflict of Interest

The authors declare no conflict of interest.

## Supporting information



Supporting Information

Supplemental Video 1

## Data Availability

The data that support the findings of this study are available in the supplementary material of this article.
