# Supplementary material for: In Situ Formation of Nanoparticles from Graft Copolypeptides Under Dispersion Polymerization Conditions
Source: Macromol Rapid Commun. 2025 Apr 28;46(14):2500069. doi: 10.1002/marc.202500069 (PMC12272521; doi:10.1002/marc.202500069)
Supplement: Supplementary file 1 — Supporting Information [file MARC-46-2500069-s002.docx]

Supporting Information

In situ formation of nanoparticles from graft copolypeptides under dispersion polymerization conditions

Ernesto Tinajero-Díaz*, Robert D. Murphy, Bo Li, Andreas Heise and Antxon Martínez de-Ilarduya

*Synthesis of the α-amino acids N-carboxyanhydride (NCAs)*. NCAs were synthesized following the literature procedures.^[1]^ γ-Benzyl-L glutamate NCA (BLG-NCA): to a round bottom flask, γ-benzyl-L glutamate (10 g, 42.14 mmol, 1.0 eq), THF (150 mL), epichlorohydrine (16.1 g, 168.6 mmol, 4 equivalent) were added sequentially under magnetic stirring. Triphosgene (6.25 g, 21.07 mmol, 0.5 eq) was added in one portion and the flask heated up under reflux. The amino acid started to disappear within 30 min; the reaction was stirred for 2 h in total and let it cool down to room temperature. Afterwards, the reaction mixture was filtered and ¾ of the THF was removed under reduced pressure. 150 mL of hexane was added to precipitate the BLG-NCA and it was put overnight in freezer at -20 °C. Thereafter, the BLG-NCA was recovered by filtration under vacuum. Then, the BLG-NCA was redissolved in 100 mL of ethyl acetate, and it was precipitated in 400 mL of hexane. This procedure was repeated twice. Finally, the purified BLG-NCA was dried under vacuum and stored at -20 °C. Yield: 87%. L-Phenylalanine NCA (Phe NCA) was synthesized following a similar fashion as for BLG-NCA.

**γ-Benzyl-L glutamate NCA (BLG-NCA)**: ^1^H NMR (300 MHz, CDCl_3_, δ, ppm). 7.42−7.32 (m, 5H), 6.55 (s, 1H), 5.15 (s, 2H), 4.39 (t, 1H), 2.61 (t, 2H), 2.34−2.07 (m, 2H).

**L-Phenylalanine NCA** (**Phe NCA)**: ^1^H NMR (300 MHz, DMSO-*d6*, δ, ppm). 9.11 (s, 1H), 7.36−7.04 (m, 5H), 4.79 (t, 1H), 3.03 (d, 2H).


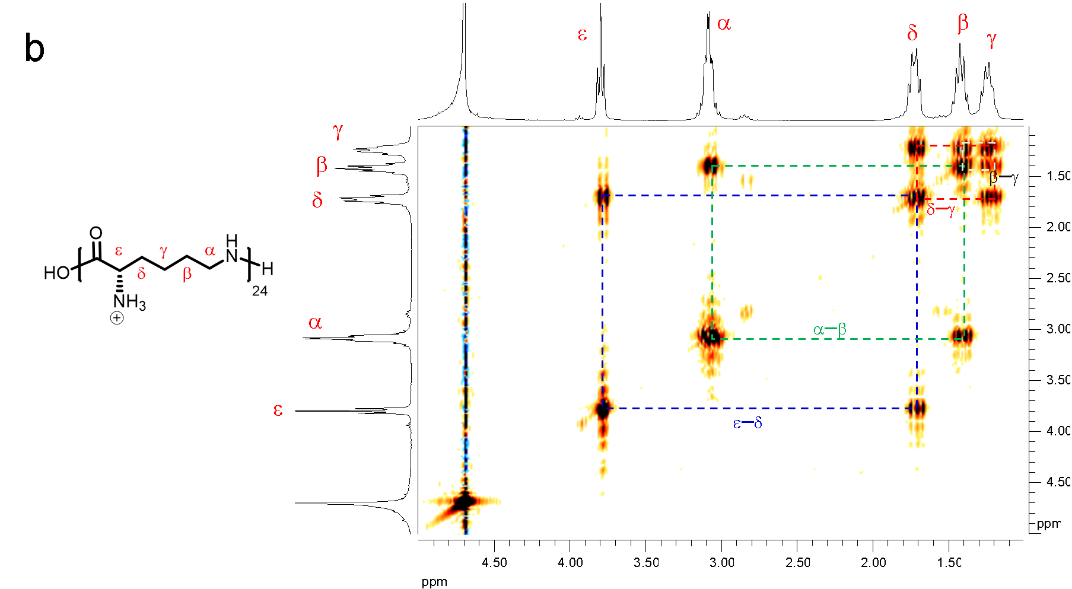


## **Figure S1**. (a) ^1^H NMR spectrum (in D_2_O) of epsilon-poly(lysine). Note: the spectrum was recorded under acidic environment, i.e., pH below its pKa, resulting in the protonation of amine groups. (b) ^1^H−^1^H COSY spectra of the εPL.

| **ACN:water**  **(% v/v)** |  | ***M*_n_ (g·mol^−1^)** | ***Đ*** |
| --- | --- | --- | --- |
| 0:100 |  | 53,262 | 1.71 |
| 25:75 |  | 58,300 | 1.26 |
| 50:50 |  | 58,900 | 1.15 |
| 75:25 |  | 51,334 | 1.21 |
| 100:0* |  | - | - |
| *The polymerization did not proceed because εPL is insoluble. | | | |

**Figure S2.** SEC traces of the ROP of BLG-NCA initiated by ePL ([NCA]/ [ePL]=6) assisted by an ACN:water solvent mixture at different v:v ratios, and average-number molecular weights (*M*_n_) and dispersities (*Đ*).

## **Figure S3.** ^1^H NMR (ACN:D_2_O (1:1 v/v)) spectra of BLG-NCA as function of time.


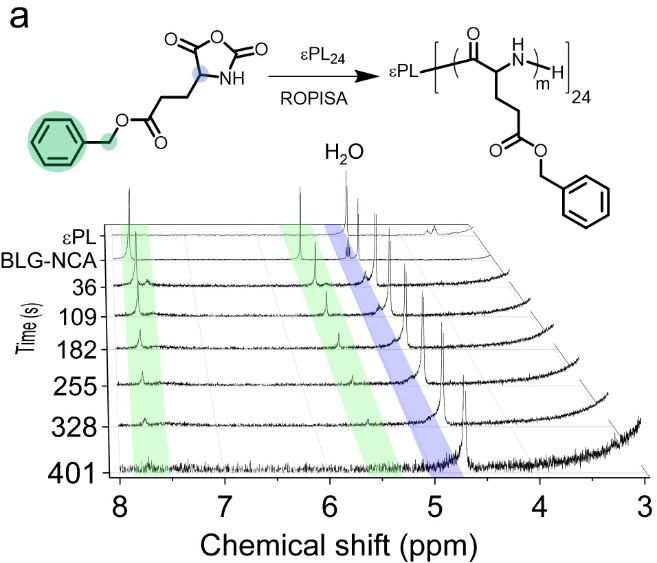





**Figure S4.** (a) ^1^H NMR (ACN:D_2_O, (1:1 v/v)) spectra exhibiting the evolution of the BLG-NCA polymerization ([NCA]/[εPL]=6). Note: εPL and BLG-NCA spectra were independently recorded for reference purposes, and (b) ¹H NMR (in CDCl₃/TFA-d (85:15% v/v)) of the εPL-g-PBLG₆ copolypeptide, obtained at different reaction times after precipitation in methanol.

**Figure S5**. DOSY ^1^H NMR of: (a) epislon-polylysine (in CDCl_3_-TFA-*d* (15:85% v/v)), and (b) εPL-*g*-PBLG_6_ copolypeptide (in CDCl_3_-TFA-*d* (85:15% v/v)).

## **Figure S6**. TEM images of the εPL-*g*-PBLG_m_ copolypeptides. Scale bar = 500 nm.

**Figure S7**. (a) DLS and (b) correlation profiles of the εPL_24_-*g*-PBLG_m_ copolypeptide nanoparticles.

**Figure S8**. ^1^H NMR spectra (in TFA-*d*) of a control experiment confirming the successful initiation of the BLG-NCA polymerization from the side-chain amine groups of the εPL. Conditions: The ROP of BLG-NCA initiated by εPL, using molar feed ratios of [NCA]/[εPL] = 0.5 and [NCA]/[εPL] = 3, was conducted in a 1:1 (v/v) ACN:water mixture at a solid content (σ_s_) of 1.5%. The NCA polymerization completed after 10 min, as confirmed by FTIR through the disappearance of the carbonyl (NCA) bands.

**Figure S9**. ^1^H NMR spectra of εPL_24_-*g*-PBLG_m_ copolypeptides in CDCl_3_ containing 10% TFA (v/v) exhibiting the displacement of the α-CH signals with temperature due to the inverse coil-to-helix transition.

**Figure S10.** (a–e) FTIR spectra of the εPL_24_-*g*-PBLG_m_ copolypeptides in the 1680−1580 cm^−1^ region, (deconvoluted curves are fitted to the α-helix (blue) and β-sheet (red) bands, respectively).

**Figure S11.** (a) SEC traces, *M*_n_ and *Đ,* and (b) ȥ-average size as function of solid content σ_s_ of the εPL_24_-*g*-PBLG_6_ copolypeptide.

**Figure S12.** (a) SEC traces and (b) DLS profiles of the ROP of L-Phenylalanine–NCA.

**Figure S13**. DOSY ^1^H–NMR of: (a) epislon-polylysine (in CDCl_3_-TFA-*d* (15:85% v/v)), and (b) εPL-*g*-PPhe_5_ copolypeptide (in CDCl_3_-TFA-*d* (85:15% v/v).

References

[1] Z.-Y. Tian, Z. Zhang, S. Wang, H. Lu, A Moisture-Tolerant Route to Unprotected α/β-Amino Acid *N*-Carboxyanhydrides and Facile Synthesis of Hyperbranched Polypeptides. Nat. Commun. **2021**, 12, 5810.
